# Supplementary material for: GWAS identifies genetic loci, lifestyle factors and circulating biomarkers that are risk factors for sarcoidosis
Source: Nat Commun. 2025 Mar 12;16:2481. doi: 10.1038/s41467-025-57829-z (PMC11903676; doi:10.1038/s41467-025-57829-z)
Supplement: Supplementary file 1 — Supplementary Information [file 41467_2025_57829_MOESM1_ESM.pdf]

Supporting information for

GWAS identifies genetic loci, lifestyle factors and circulating biomarkers that are risk factors for sarcoidosis

Shuai Yuan, Jie Chen, Jiawei Geng, Sizheng Steven Zhao, James Yarmolinsky, Elizabeth V. Arkema, Sarah Abramowitz, Michael G Levin, Kostas K Tsilidis, Stephen Burgess, Scott M. Damrauer, Susanna C. Larsson

Table of Contents

Supplementary methods 2

Supplementary figure 1. QQ-plot of genome-wide associations in all participants (a), Europeans (b), and African Americans (c). 3

Supplementary figure 2. Genome-wide association with sarcoidosis. a. associations in all participants. b. associations in Europeans. c. associations in African Americans. 4

Supplementary figure 3. Locus plot of loci 1-8 associated with sarcoidosis. 5

Supplementary figure 4. Locus plot of loci 9-16 associated with sarcoidosis. 6

Supplementary figure 5. Locus plot of loci 17-24 associated with sarcoidosis. 7

Supplementary figure 6. Locus plot of loci 25-28 associated with sarcoidosis. 8

Supplementary figure 7. Scatter plot of identified loci. 9

Supplementary figure 8. Tissue-specific enrichment using FUMA. 10

Supplementary methods

Traditional colocalization analysis

We conducted colocalization analysis using the coloc R package to test whether identified associations between inflammatory markers and sarcoidosis were driven by linkage disequilibrium. For each locus, the Bayesian method assessed the support for the following five exclusive hypotheses: 1) no association with either trait; 2) association with trait 1 only; 3) association with trait 2 only; 4) both traits are associated, but distinct causal variants were for two traits; and 5) both traits are associated, and the same shares causal variant for both traits. The analysis provides posterior probabilities for each hypothesis testing (H0, H1, H2, H3, and H4). We set prior probabilities of the SNP being associated with trait 1 only (p1) at 1×10^{-4}; the probability of the SNP being associated with trait 2 only (p2) at 1×10^{-4}; and the probability of the SNP being associated with both traits (p12) at 1×10^{-5} two signals were considered to have strong evidence of colocalization if the posterior probability for shared causal variants (PH4) was ≥0.7.

SuSiE (Sum of Single Effects) colocalization

SuSiE is a computational method used in genomics and statistical genetics for colocalization analysis. SuSiE utilizes statistical models and Bayesian inference techniques to assess whether genetic variants associated with one trait are likely to be shared with those associated with another trait. By examining the overlap of genetic signals, SuSiE provides insights into whether multiple traits may have common underlying genetic factors, thereby facilitating the discovery of shared genetic mechanisms or pathways across different phenotypes. SuSiE offers strengths compared with traditional genetic association analysis by identifying shared genetic signals between different traits, reducing false positives, providing insights into common biological mechanisms, and exceling at handling more than one signals within one gene region.

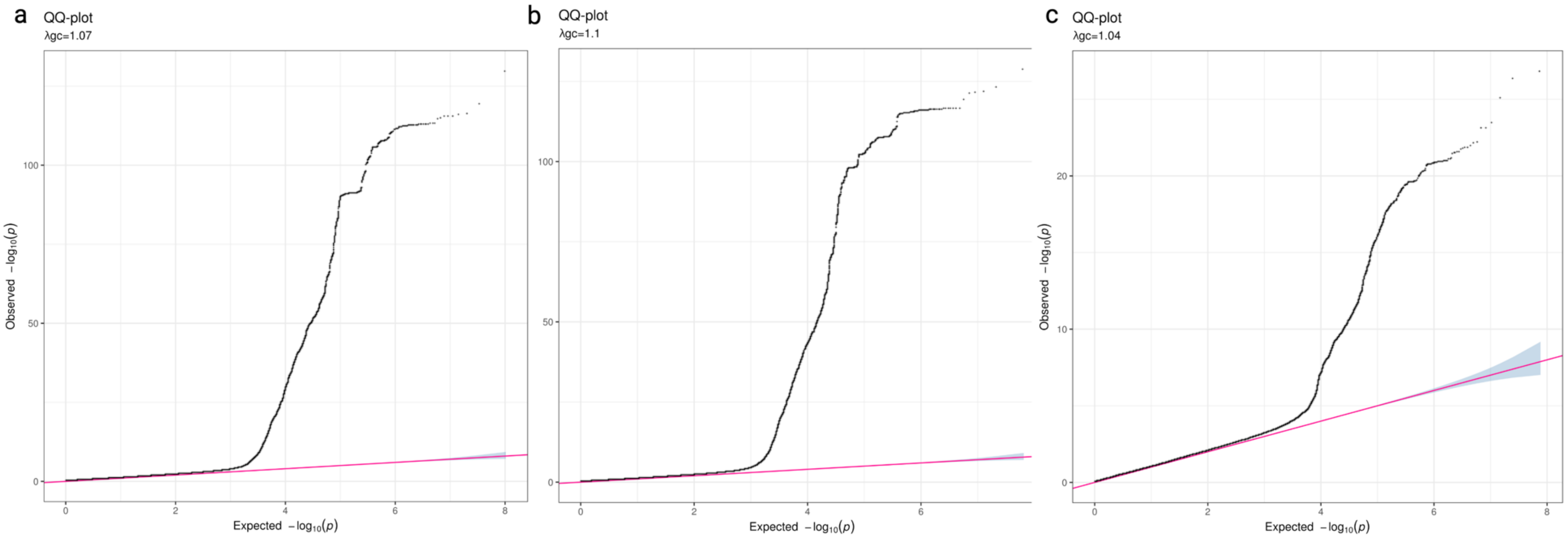

Supplementary figure 1. QQ-plot of genome-wide associations in all participants (a), Europeans (b), and African Americans (c).

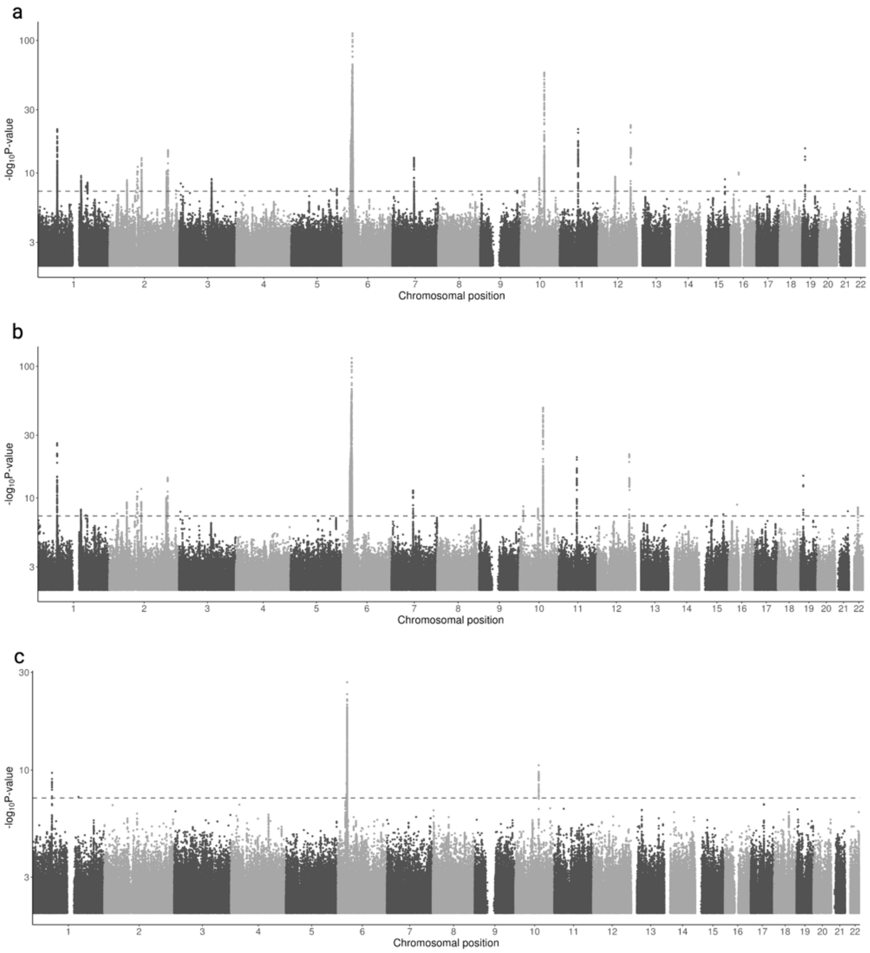

Supplementary figure 2. Genome-wide association with sarcoidosis. a. associations in all participants. b. associations in Europeans. c. associations in African Americans.

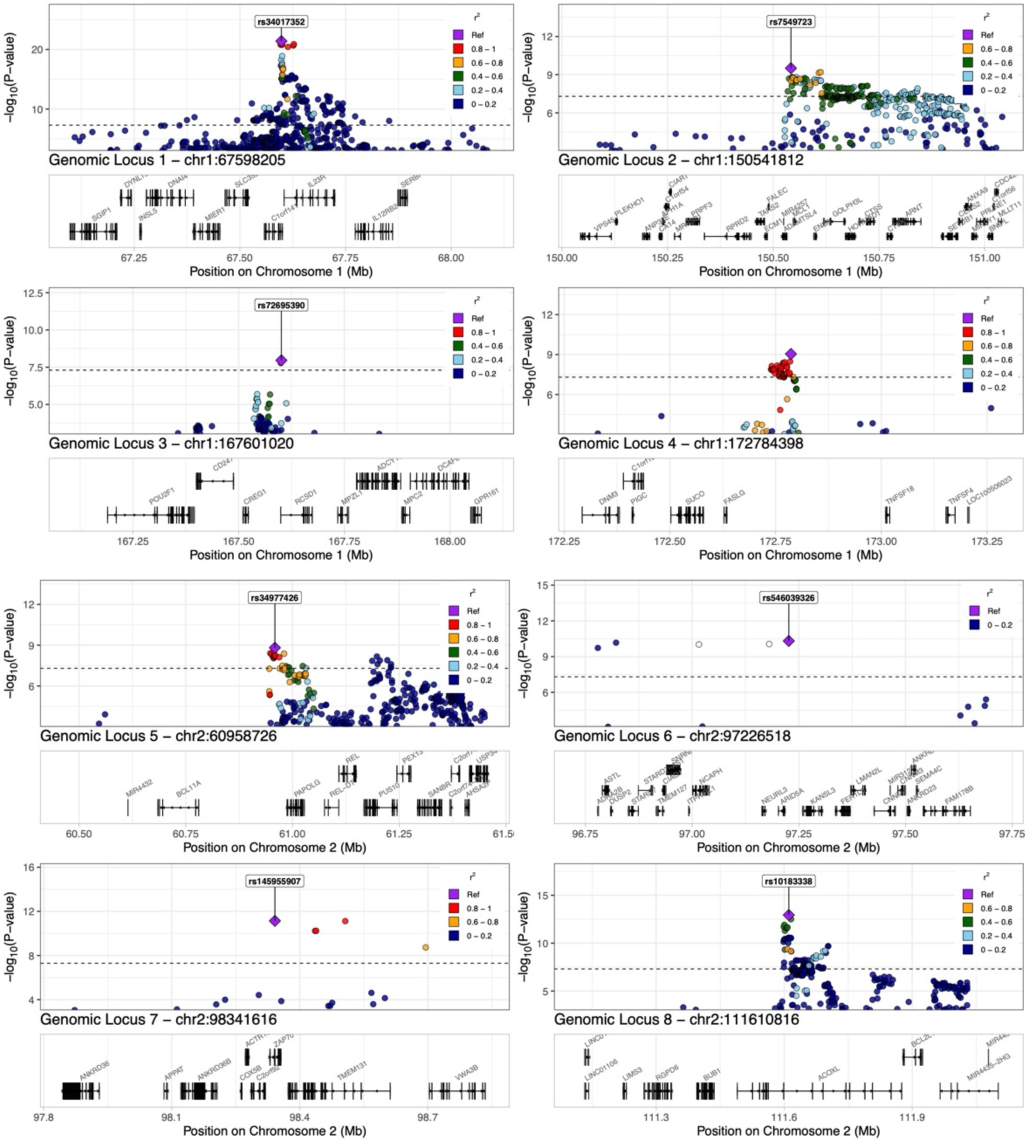

Supplementary figure 3. Locus plot of loci 1-8 associated with sarcoidosis.

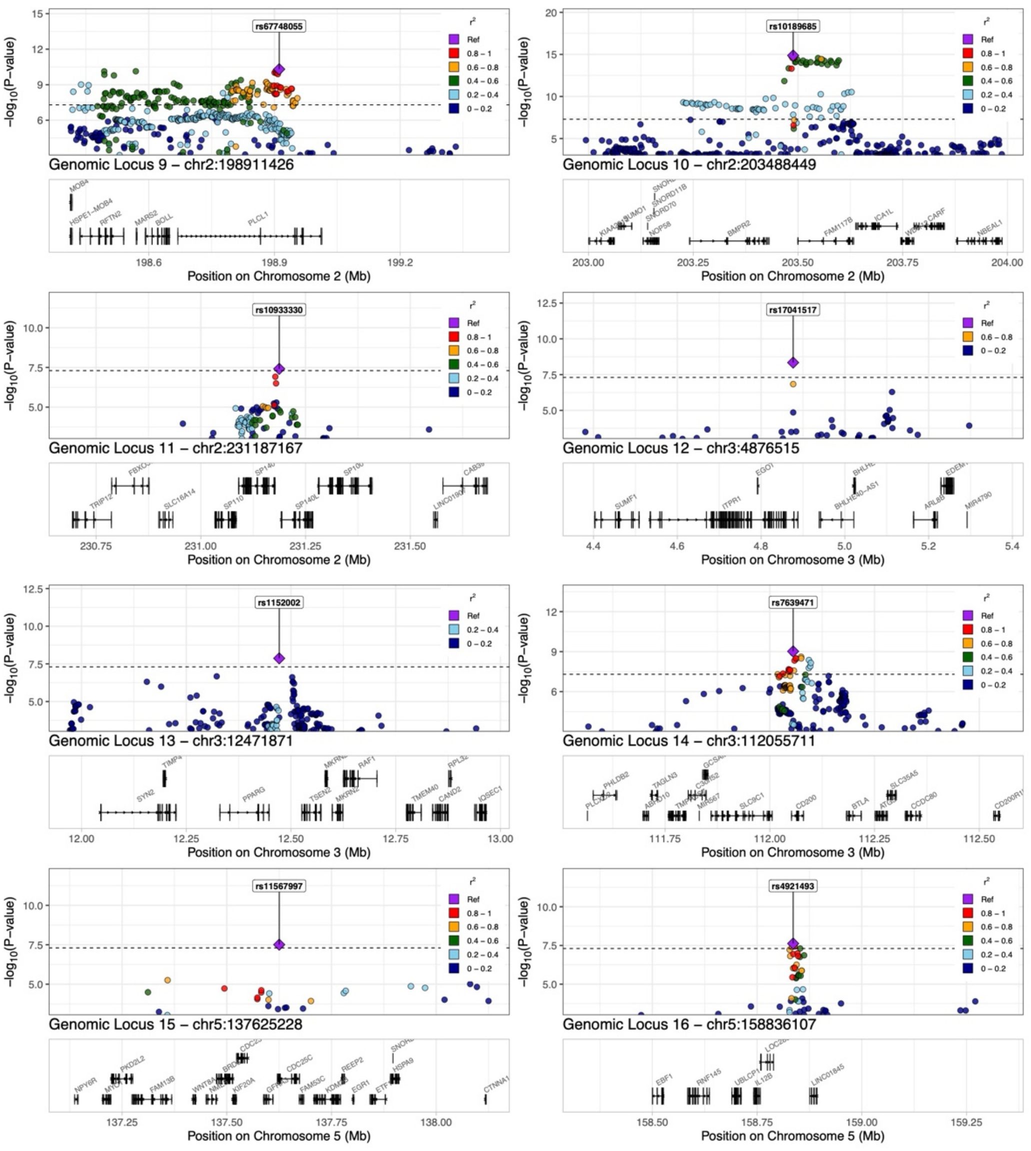

Supplementary figure 4. Locus plot of loci 9-16 associated with sarcoidosis.

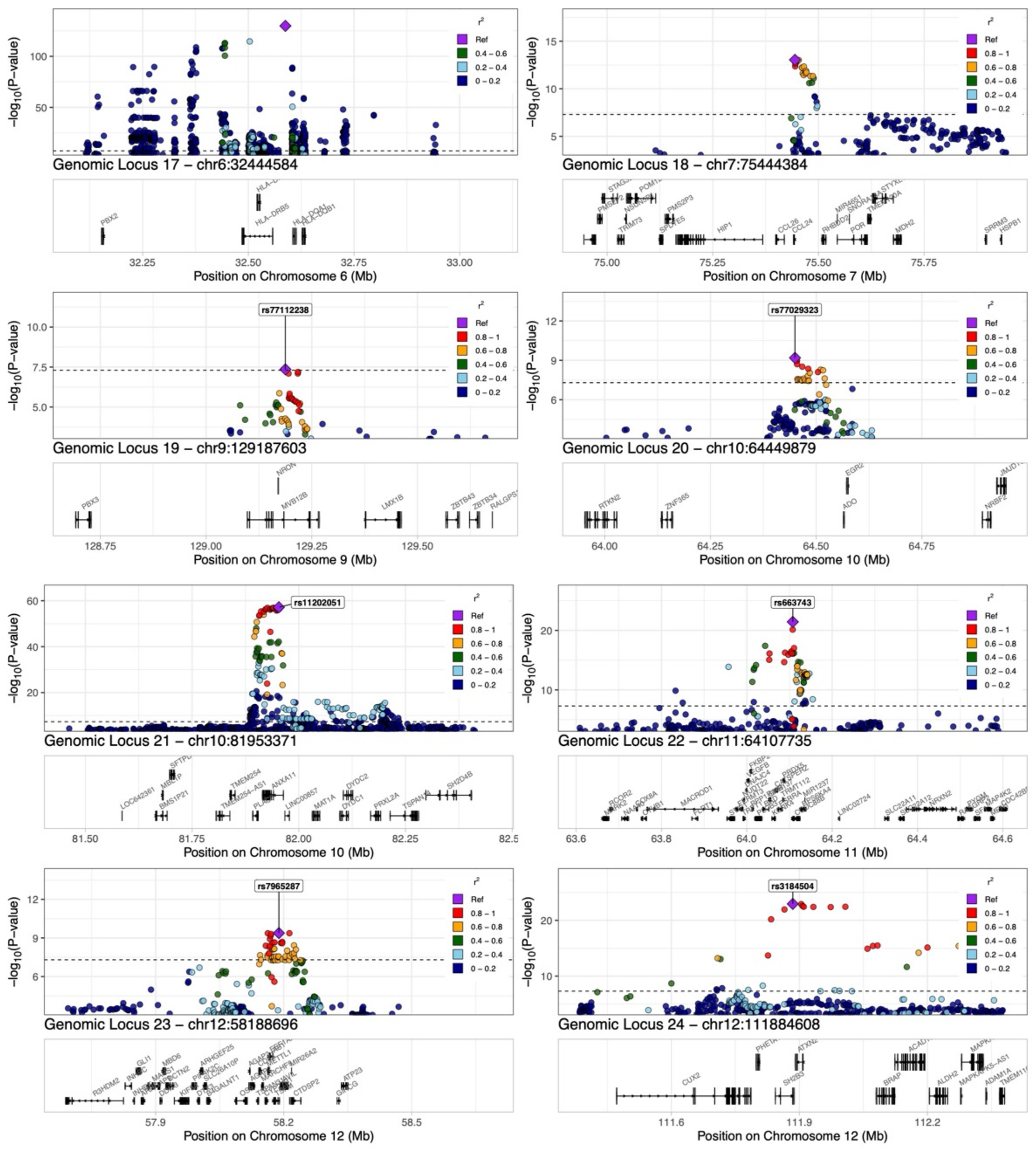

Supplementary figure 5. Locus plot of loci 17-24 associated with sarcoidosis.

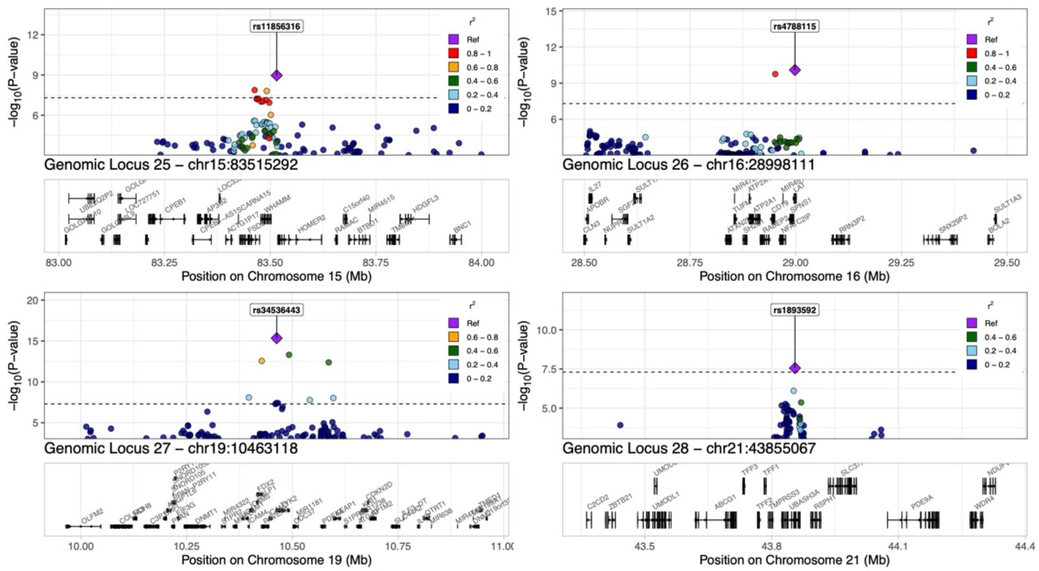

Supplementary figure 6. Locus plot of loci 25-28 associated with sarcoidosis.

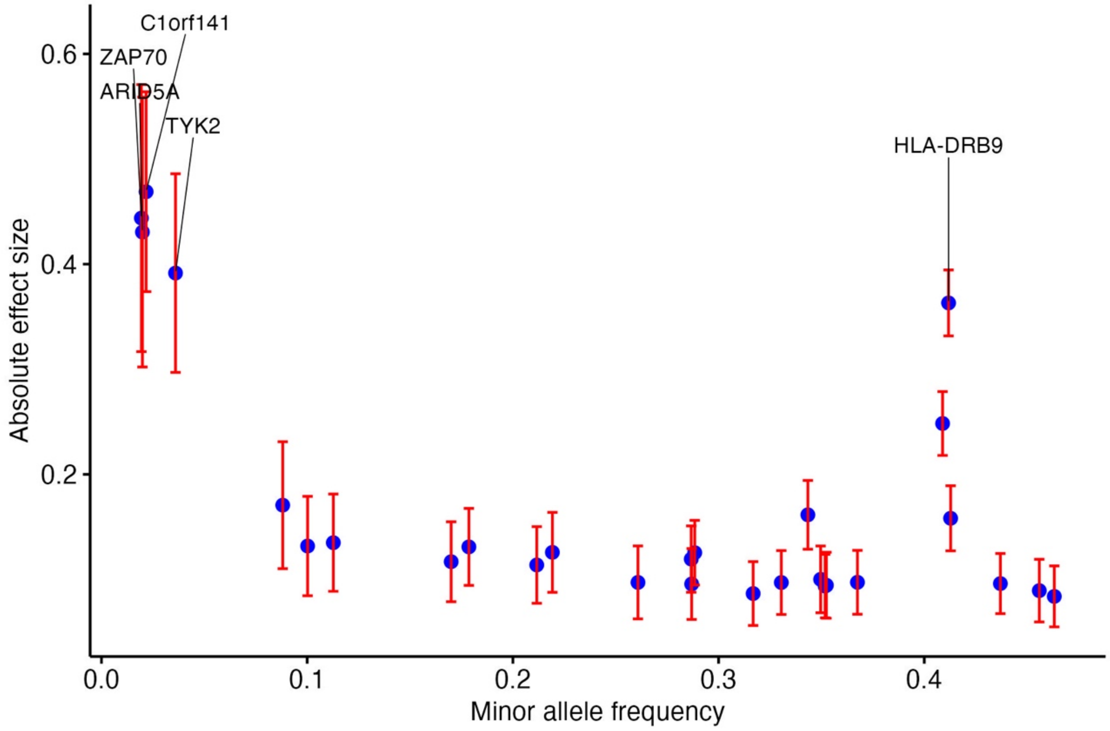

Supplementary figure 7. Scatter plot of identified loci.

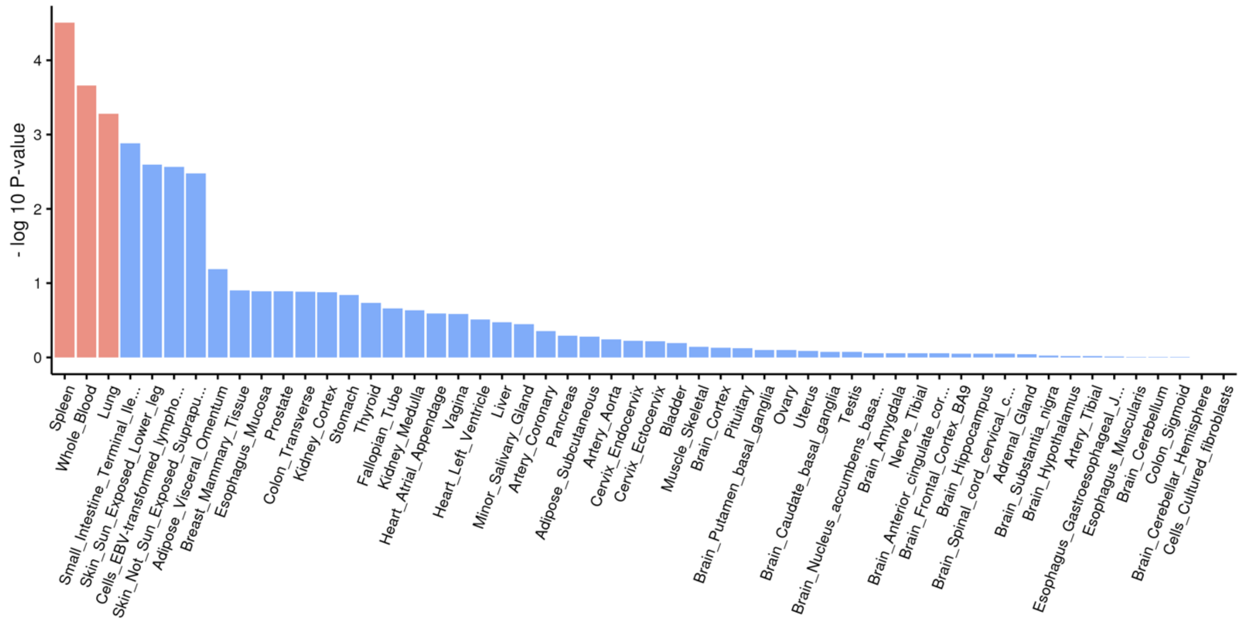

Supplementary figure 8. Tissue-specific enrichment using FUMA.
