# Supplementary material for: GWAS identifies genetic loci, lifestyle factors and circulating biomarkers that are risk factors for sarcoidosis
Source: Nat Commun. 2025 Mar 12;16:2481. doi: 10.1038/s41467-025-57829-z (PMC11903676; doi:10.1038/s41467-025-57829-z)
Supplement: Supplementary file 2 — Description of Additional Supplementary Files [file 41467_2025_57829_MOESM2_ESM.pdf]

Description of Additional Supplementary Files

File name: Supplementary Data 1

Description: Phenotypes associated with sarcoidosis-related loci in GWAS Catalog.

File name: Supplementary Data 2

Description: Pleiotropic associations with autoimmune diseases for 28 sarcoidosis-associated loci in the GWAS Catalog database.

File name: Supplementary Data 3

Description: Results of TWAS in spleen, whole blood, and lung based on GTEx v8 data.

File name: Supplementary Data 4

Description: Mendelian randomization association between genetically predicted 65 inflammatory markers and risk of sarcoidosis.

File name: Supplementary Data 5

Description: Genetic colocalization evidence of the association between inflammatory markers and sarcoidosis.

File name: Supplementary Data 6

Description: Associations between genetically predicted proteins and risk of sarcoidosis in the Fenland study as replication.

File name: Supplementary Data 7

Description: Data sources for exposures used in the Mendelian randomization analysis.
